# Supplementary material for: Scientific publications that use promotional language in the abstract receive more citations and public attention
Source: Commun Psychol. 2025 Aug 5;3:118. doi: 10.1038/s44271-025-00293-8 (PMC12325648; doi:10.1038/s44271-025-00293-8)
Supplement: Supplementary file 2 — Supplementary Information [file 44271_2025_293_MOESM2_ESM.docx]

**Scientific publications that use promotional language receive more citations and social media mentions**

**Supplementary Information File**

Olga Stavrova

University of Lübeck (Germany) and Tilburg University (Netherlands)

Bennett Kleinberg

Tilburg University (Netherlands) and University College London (United Kingdom)

Anthony M. Evans

Allstate Corporation (United States of America)

Milena Ivanović

Ipsos Strategic Marketing (Serbia)

Contents

[Promotional language dictionary 2](#_Toc198458203)

[Main variables distributions 3](#_Toc198458204)

[Robustness checks 4](#_Toc198458205)

[Promotional language and impact over time 22](#_Toc198458206)

[Correlations among impact indicators 25](#_Toc198458207)

[List of the subject fields 26](#_Toc198458208)

[References 27](#_Toc198458209)

# Promotional language dictionary

The dictionary includes the following words^1^:

"compelling", "critical", "crucial", "essential", "foundational" , "fundamental", "imperative" , "important" , "indispensable" , "invaluable" , "key" , "major" , "paramount" , "pivotal" , "significant" , "strategic" , "timely" , "ultimate" , "urgent" , "vital" , "creative" , "emerging" , "first" , "groundbreaking" , "innovative" , "latest", "novel" , "revolutionary" , "unique" , "unparalleled" , "unprecedented" , "accurate" , "advanced" , "careful" , "cohesive" , "detailed" , "nuanced" , "powerful" , "quality" , "reproducible" , "rigorous" , "robust" , "scientific" , "sophisticated" , "strong" , "systematic" , "accessible" , "actionable" , "deployable" , "durable" , "easy" , "effective" , "efficacious" , "efficient" , "generalizable" , "ideal" , "impactful" , "intuitive" , "meaningful" , "productive" , "ready" , "relevant" , "rich" , "safer" , "scalable" , "seamless" , "sustainable" , "synergistic" , "tailored" , "tangible" , "transformative" , "user-friendly" , "ambitious" , "collegial" , "dedicated" , "exceptional" , "experienced" , "intellectual" , "longstanding" , "motivated" , "premier" , "prestigious" , "promising" , "qualified" , "renowned" , "senior" , "skilled" , "stellar" , "successful", "talented", "vibrant", "ample", "biggest", "broad", "comprehensive", "considerable", "deeper", "diverse", "enormous", "expansive", "extensive", "fastest", "greatest", "huge", "immediate", "immense", "interdisciplinary", "international", "interprofessional", "largest", "massive" , "multidisciplinary" , "myriad" , "overwhelming”, "substantial", "top" , "transdisciplinary" , "tremendous" , "vast" , "attractive" , "confident" , "exciting", "incredible", "interesting", "intriguing", "notable", "outstanding", "remarkable", "surprising", "alarming", "daunting", "desperate", "devastating", "dire", "dismal", "elusive", "stark", "unanswered", "unmet".

# Main variables distributions

Supplementary Figure 1

**
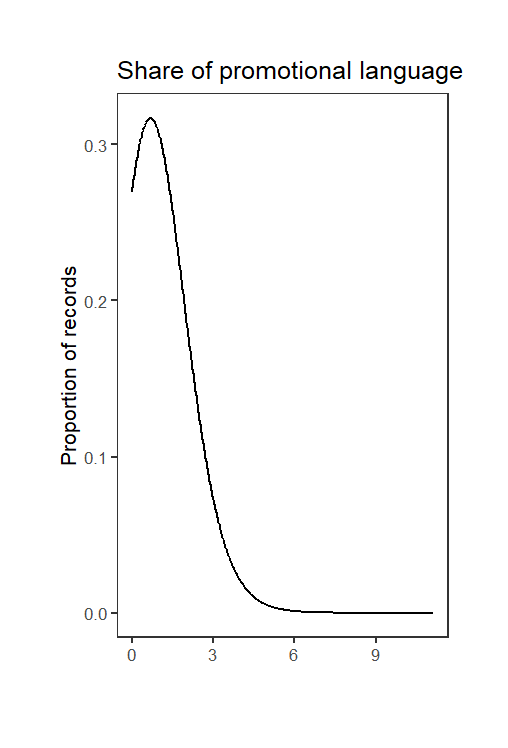

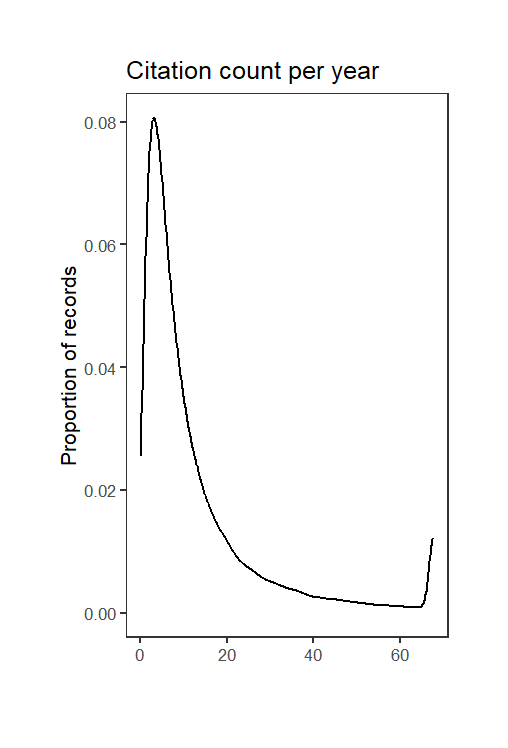

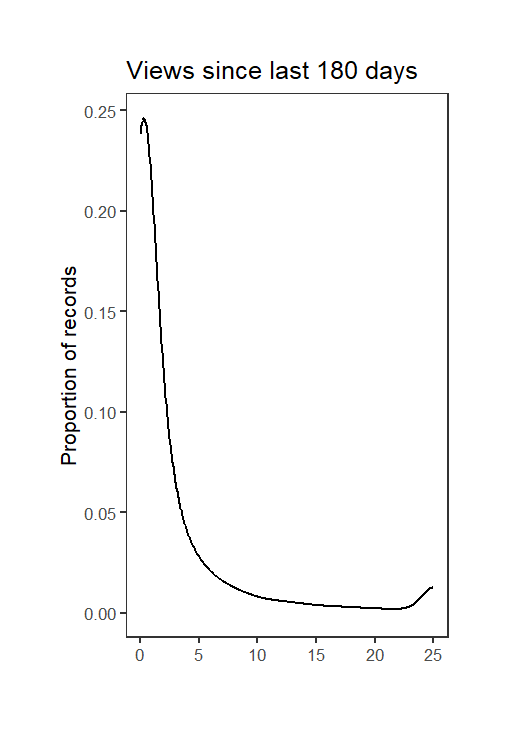

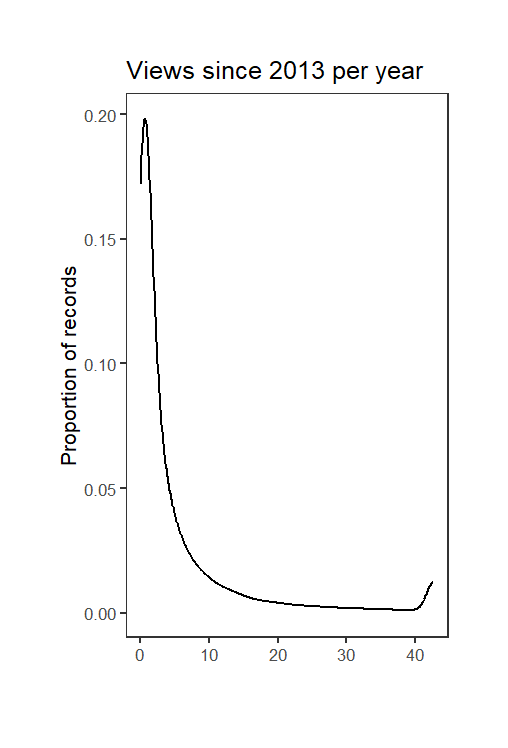

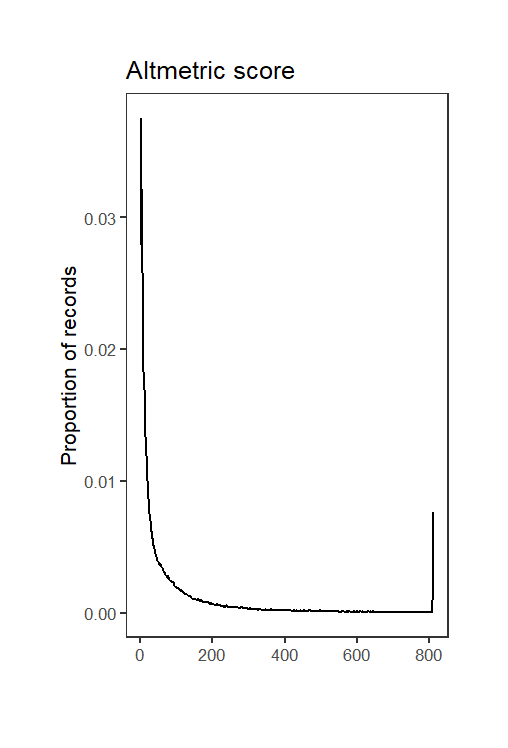

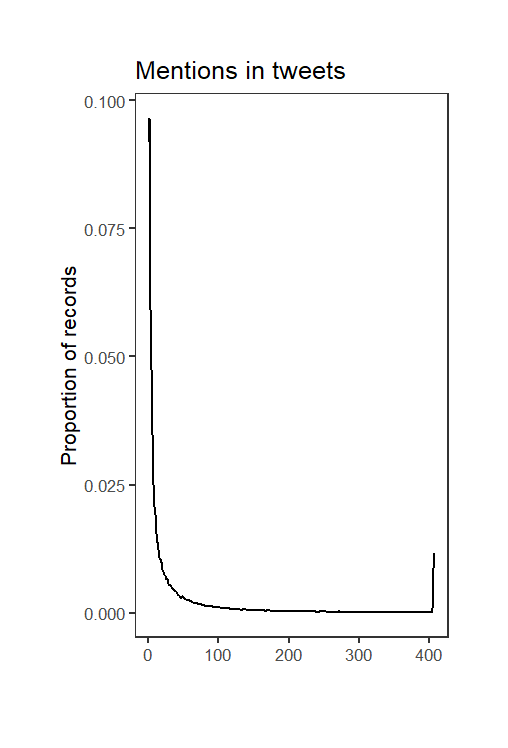

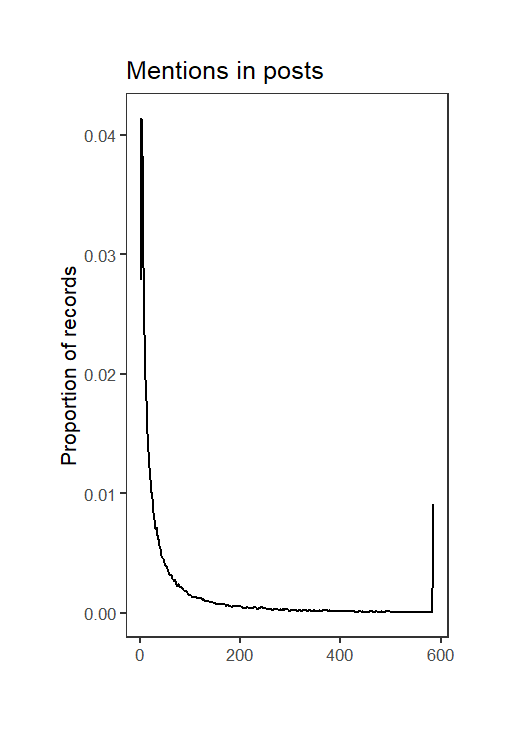
**

*Note.* All variables were winsorized for visualization (the top 3%) but were not transformed in the analyses.

# Robustness checks

We tested the following alternative models:

1) as Altmetric started collecting data in 2011, we restricted the analyses of public attention to the papers published after 2010 (Supplementary Tables 1-3)

2) we excluded abstracts with an exceptionally high (3 SD above the mean) share of promotional words (Supplementary Table 4-6).

3) as the Web of Science database was accessed in June 2023, we restricted the analyses to the papers that were published before 2023 (Supplementary Tables 7-9).

Supplementary Table 1

*Promotional language and public attention in papers published after 2010*

|  | **Altmetric** | | | **Tweets** | | | **Posts** | | |
| --- | --- | --- | --- | --- | --- | --- | --- | --- | --- |
| *Predictors* | *b* | *CI* | *p* | *IRR* | *CI* | *p* | *IRR* | *CI* | *p* |
| (Intercept) | 1.48 | 1.47 – 1.49 | **<0.001** | 86.29 | 84.42 – 88.21 | **<0.001** | 121.52 | 119.00 – 124.09 | **<0.001** |
| Promotional language | 0.04 | 0.04 – 0.05 | **<0.001** | 1.08 | 1.06 – 1.10 | **<0.001** | 1.08 | 1.06 – 1.10 | **<0.001** |
| Observations | 49520 | | | 49520 | | | 49520 | | |
|  | **Altmetric** | | | **Tweets** | | | **Posts** | | |
| *Predictors* | *b* | *CI* | *p* | *IRR* | *CI* | *p* | *IRR* | *CI* | *p* |
| (Intercept) | 0.13 | 0.06 – 0.19 | **<0.001** | 0.24 | 0.21 – 0.27 | **<0.001** | 1.11 | 0.98 – 1.25 | 0.090 |
| Promotional language | 0.04 | 0.03 – 0.05 | **<0.001** | 1.06 | 1.05 – 1.08 | **<0.001** | 1.07 | 1.06 – 1.09 | **<0.001** |
| Journal [nature] | 0.91 | 0.89 – 0.93 | **<0.001** | 4.04 | 3.92 – 4.17 | **<0.001** | 3.98 | 3.86 – 4.10 | **<0.001** |
| Journal [science] | 0.96 | 0.94 – 0.98 | **<0.001** | 4.28 | 4.12 – 4.46 | **<0.001** | 4.26 | 4.10 – 4.43 | **<0.001** |
| Year | 0.05 | 0.05 – 0.05 | **<0.001** | 1.24 | 1.24 – 1.25 | **<0.001** | 1.18 | 1.18 – 1.19 | **<0.001** |
| Word count | 0.00 | 0.00 – 0.00 | **<0.001** | 1.00 | 1.00 – 1.00 | **<0.001** | 1.00 | 1.00 – 1.00 | **<0.001** |
| Field [formal & natural] | -0.65 | -0.68 – -0.62 | **<0.001** | 0.21 | 0.19 – 0.22 | **<0.001** | 0.24 | 0.23 – 0.26 | **<0.001** |
| Field [medical & health] | -0.53 | -0.56 – -0.50 | **<0.001** | 0.35 | 0.33 – 0.37 | **<0.001** | 0.40 | 0.38 – 0.42 | **<0.001** |
| Author number | 0.00 | 0.00 – 0.00 | **<0.001** | 1.01 | 1.01 – 1.01 | **<0.001** | 1.01 | 1.01 – 1.01 | **<0.001** |
| Institutional diversity | 0.11 | 0.09 – 0.12 | **<0.001** | 1.38 | 1.34 – 1.42 | **<0.001** | 1.37 | 1.34 – 1.41 | **<0.001** |
| Positivity | 0.00 | 0.00 – 0.00 | **<0.001** | 1.00 | 1.00 – 1.00 | **0.041** | 1.00 | 1.00 – 1.00 | 0.105 |
| Observations | 49517 | | | 49517 | | | 49517 | | |

*Note*. *b* = unstandardized regression coefficient; IRR=Incidence Rate Ratio; CI = 95% Confidence Interval. Citations: citation count per year; Views 180: the number of times the record has been accessed on the Web of Science platform (e.g. by clicking on the link to the publisher’s website or by saving it in a bibliographic management tool) in the last 180 days; Views 2013: the number of times the record has been accessed on the Web of Science platform (e.g. by clicking on the link to the publisher’s website or by saving it in a bibliographic management tool) since 2013, per year. Altmetric: Altmetric score; Tweets: number of mentions on Twitter/X; Posts: overall number of mentions in any type of online document (news, social media, policy documents etc.). Promotional language: share of promotional language in the abstract; Journal: PNAS is reference category; Year: publication year; Word count: number of words in the abstract; Field: social and humanities is reference category; author number: number of authors; institutional diversity: number of listed affiliations divided by the number of authors, higher score indicates more collaborations across different institutions; Positivity: overall semantic positivity of the abstract, obtained using the Linguistic Inquiry and Word Count software (LIWC, 2007).

Supplementary Table 2

*Effect of promotional language on public attention moderated by first author gender in papers published after 2010*

|  | **Altmetric** | | | **Tweets** | | | **Posts** | | |
| --- | --- | --- | --- | --- | --- | --- | --- | --- | --- |
| *Predictors* | *b* | *CI* | *p* | *IRR* | *CI* | *p* | *IRR* | *CI* | *p* |
| (Intercept) | 1.54 | 1.52 – 1.55 | **<0.001** | 99.49 | 96.17 – 102.93 | **<0.001** | 139.10 | 134.67 – 143.67 | **<0.001** |
| Promotional language | 0.05 | 0.04 – 0.06 | **<0.001** | 1.09 | 1.06 – 1.12 | **<0.001** | 1.09 | 1.06 – 1.12 | **<0.001** |
| First author gender | -0.09 | -0.12 – -0.06 | **<0.001** | 0.91 | 0.85 – 0.96 | **0.001** | 0.91 | 0.86 – 0.96 | **0.001** |
| Promotional language * First author gender | 0.01 | -0.02 – 0.03 | 0.644 | 0.97 | 0.93 – 1.02 | 0.188 | 0.96 | 0.92 – 1.00 | 0.068 |
| Observations | 30755 | | | 30755 | | | 30755 | | |
|  | **Altmetric** | | | **Tweets** | | | **Posts** | | |
| *Predictors* | *b* | *CI* | *p* | *IRR* | *CI* | *p* | *IRR* | *CI* | *p* |
| (Intercept) | -0.03 | -0.10 – 0.05 | 0.534 | 0.28 | 0.24 – 0.33 | **<0.001** | 1.21 | 1.04 – 1.40 | **0.013** |
| Promotional language | 0.05 | 0.04 – 0.06 | **<0.001** | 1.09 | 1.07 – 1.12 | **<0.001** | 1.09 | 1.07 – 1.11 | **<0.001** |
| First author gender | -0.04 | -0.06 – -0.01 | **0.008** | 0.97 | 0.93 – 1.02 | 0.288 | 0.93 | 0.89 – 0.98 | **0.004** |
| Journal [nature] | 0.86 | 0.84 – 0.88 | **<0.001** | 3.96 | 3.81 – 4.13 | **<0.001** | 3.90 | 3.76 – 4.06 | **<0.001** |
| Journal [science] | 0.93 | 0.90 – 0.96 | **<0.001** | 4.17 | 3.96 – 4.38 | **<0.001** | 4.15 | 3.96 – 4.36 | **<0.001** |
| Year | 0.06 | 0.06 – 0.06 | **<0.001** | 1.25 | 1.24 – 1.25 | **<0.001** | 1.19 | 1.18 – 1.19 | **<0.001** |
| Word count | 0.00 | 0.00 – 0.00 | **<0.001** | 1.00 | 1.00 – 1.00 | **<0.001** | 1.00 | 1.00 – 1.00 | **<0.001** |
| Field [formal & natural] | -0.61 | -0.64 – -0.57 | **<0.001** | 0.21 | 0.20 – 0.23 | **<0.001** | 0.25 | 0.24 – 0.27 | **<0.001** |
| Field [medical & health] | -0.50 | -0.53 – -0.46 | **<0.001** | 0.35 | 0.33 – 0.38 | **<0.001** | 0.40 | 0.38 – 0.43 | **<0.001** |
| Author number | 0.00 | 0.00 – 0.00 | **<0.001** | 1.01 | 1.01 – 1.01 | **<0.001** | 1.01 | 1.01 – 1.01 | **<0.001** |
| Institutional diversity | 0.09 | 0.07 – 0.10 | **<0.001** | 1.24 | 1.20 – 1.28 | **<0.001** | 1.25 | 1.21 – 1.29 | **<0.001** |
| Positivity | 0.00 | 0.00 – 0.00 | **<0.001** | 1.00 | 1.00 – 1.00 | **0.003** | 1.00 | 1.00 – 1.00 | **0.010** |
| Promotional language * First author gender | 0.01 | -0.01 – 0.03 | 0.507 | 0.91 | 0.87 – 0.94 | **<0.001** | 0.94 | 0.90 – 0.97 | **<0.001** |
| Observations | 30755 | | | 30755 | | | 30755 | | |

*Note*. *b* = unstandardized regression coefficient; IRR=Incidence Rate Ratio; CI = 95% Confidence Interval. Citations: citation count per year; Views 180: the number of times the record has been accessed on the Web of Science platform (e.g. by clicking on the link to the publisher’s website or by saving it in a bibliographic management tool) in the last 180 days; Views 2013: the number of times the record has been accessed on the Web of Science platform (e.g. by clicking on the link to the publisher’s website or by saving it in a bibliographic management tool) since 2013, per year. Altmetric: Altmetric score; Tweets: number of mentions on Twitter/X; Posts: overall number of mentions in any type of online document (news, social media, policy documents etc.). First author gender: 1=female, 0=male. Promotional language: share of promotional language in the abstract; Journal: PNAS is reference category; Year: publication year; Word count: number of words in the abstract; Field: social and humanities is reference category; author number: number of authors; institutional diversity: number of listed affiliations divided by the number of authors, higher score indicates more collaborations across different institutions; Positivity: overall semantic positivity of the abstract, obtained using the Linguistic Inquiry and Word Count software (LIWC, 2007).

Supplementary Table 3

*Effect of promotional language on public attention moderated by last author gender in papers published after 2010*

|  | **Altmetric** | | | **Tweets** | | | **Posts** | | |
| --- | --- | --- | --- | --- | --- | --- | --- | --- | --- |
| *Predictors* | *b* | *CI* | *p* | *IRR* | *CI* | *p* | *IRR* | *CI* | *p* |
| (Intercept) | 1.47 | 1.46 – 1.49 | **<0.001** | 91.62 | 89.06 – 94.25 | **<0.001** | 128.01 | 124.60 – 131.52 | **<0.001** |
| Promotional language | 0.06 | 0.05 – 0.07 | **<0.001** | 1.09 | 1.06 – 1.11 | **<0.001** | 1.09 | 1.07 – 1.11 | **<0.001** |
| Last author gender | 0.02 | -0.01 – 0.06 | 0.229 | 0.98 | 0.92 – 1.05 | 0.633 | 0.98 | 0.92 – 1.04 | 0.458 |
| Promotional language * Last author gender | -0.04 | -0.07 – -0.01 | **0.002** | 0.96 | 0.91 – 1.01 | 0.085 | 0.96 | 0.91 – 1.00 | 0.076 |
| Observations | 36468 | | | 36468 | | | 36468 | | |
|  | **Altmetric** | | | **Tweets** | | | **Posts** | | |
| *Predictors* | *b* | *CI* | *p* | *IRR* | *CI* | *p* | *IRR* | *CI* | *p* |
| (Intercept) | 0.03 | -0.04 – 0.10 | 0.432 | 0.16 | 0.14 – 0.18 | **<0.001** | 0.77 | 0.67 – 0.89 | **<0.001** |
| Promotional language | 0.05 | 0.04 – 0.06 | **<0.001** | 1.08 | 1.06 – 1.10 | **<0.001** | 1.09 | 1.07 – 1.11 | **<0.001** |
| Last author gender | 0.05 | 0.02 – 0.08 | **<0.001** | 0.92 | 0.87 – 0.97 | **0.002** | 0.94 | 0.90 – 1.00 | **0.035** |
| Journal [nature] | 0.87 | 0.85 – 0.89 | **<0.001** | 4.05 | 3.90 – 4.20 | **<0.001** | 3.92 | 3.78 – 4.06 | **<0.001** |
| Journal [science] | 0.93 | 0.91 – 0.96 | **<0.001** | 4.52 | 4.31 – 4.73 | **<0.001** | 4.37 | 4.18 – 4.57 | **<0.001** |
| Year | 0.05 | 0.05 – 0.06 | **<0.001** | 1.26 | 1.26 – 1.27 | **<0.001** | 1.20 | 1.19 – 1.20 | **<0.001** |
| Word count | 0.00 | 0.00 – 0.00 | **<0.001** | 1.00 | 1.00 – 1.00 | **<0.001** | 1.00 | 1.00 – 1.00 | **<0.001** |
| Field [formal & natural] | -0.62 | -0.66 – -0.59 | **<0.001** | 0.22 | 0.20 – 0.23 | **<0.001** | 0.25 | 0.23 – 0.26 | **<0.001** |
| Field [medical & health] | -0.52 | -0.56 – -0.48 | **<0.001** | 0.35 | 0.32 – 0.37 | **<0.001** | 0.39 | 0.36 – 0.41 | **<0.001** |
| Author number | 0.00 | 0.00 – 0.00 | **<0.001** | 1.01 | 1.01 – 1.01 | **<0.001** | 1.01 | 1.01 – 1.01 | **<0.001** |
| Institutional diversity | 0.09 | 0.07 – 0.11 | **<0.001** | 1.38 | 1.34 – 1.43 | **<0.001** | 1.37 | 1.33 – 1.41 | **<0.001** |
| Positivity | 0.00 | 0.00 – 0.00 | **<0.001** | 1.00 | 1.00 – 1.00 | **0.001** | 1.00 | 1.00 – 1.00 | **0.017** |
| Promotional language * Last author gender | -0.03 | -0.05 – -0.01 | **0.006** | 0.96 | 0.92 – 1.01 | 0.088 | 0.96 | 0.92 – 1.00 | 0.071 |
| Observations | 36468 | | | 36468 | | | 36468 | | |

*Note*. *b* = unstandardized regression coefficient; IRR=Incidence Rate Ratio; CI = 95% Confidence Interval. Citations: citation count per year; Views 180: the number of times the record has been accessed on the Web of Science platform (e.g. by clicking on the link to the publisher’s website or by saving it in a bibliographic management tool) in the last 180 days; Views 2013: the number of times the record has been accessed on the Web of Science platform (e.g. by clicking on the link to the publisher’s website or by saving it in a bibliographic management tool) since 2013, per year. Altmetric: Altmetric score; Tweets: number of mentions on Twitter/X; Posts: overall number of mentions in any type of online document (news, social media, policy documents etc.). Last author gender: 1=female, 0=male. Promotional language: share of promotional language in the abstract; Journal: PNAS is reference category; Year: publication year; Word count: number of words in the abstract; Field: social and humanities is reference category; author number: number of authors; institutional diversity: number of listed affiliations divided by the number of authors, higher score indicates more collaborations across different institutions; Positivity: overall semantic positivity of the abstract, obtained using the Linguistic Inquiry and Word Count software (LIWC, 2007).

Supplementary Table 4

*Promotional language, academic impact and public attention, without outliers^a^*

|  | **Citations** | | | **Views 180** | | | **Views 2013** | | | **Altmetric** | | | **Tweets** | | | **Posts** | | |
| --- | --- | --- | --- | --- | --- | --- | --- | --- | --- | --- | --- | --- | --- | --- | --- | --- | --- | --- |
| *Predictors* | *IRR* | *CI* | *p* | *IRR* | *CI* | *p* | *IRR* | *CI* | *p* | *b* | *CI* | *p* | *IRR* | *CI* | *p* | *IRR* | *CI* | *p* |
| (Intercept) | 13.16 | 13.05 – 13.27 | **<0.001** | 2.80 | 2.75 – 2.85 | **<0.001** | 5.14 | 5.07 – 5.20 | **<0.001** | 1.37 | 1.36 – 1.38 | **<0.001** | 65.69 | 64.34 – 67.06 | **<0.001** | 98.53 | 96.69 – 100.40 | **<0.001** |
| Promotional language | 1.15 | 1.15 – 1.16 | **<0.001** | 1.40 | 1.38 – 1.43 | **<0.001** | 1.40 | 1.39 – 1.42 | **<0.001** | 0.06 | 0.05 – 0.07 | **<0.001** | 1.12 | 1.10 – 1.14 | **<0.001** | 1.11 | 1.09 – 1.13 | **<0.001** |
| Observations | 134919 | | | 134919 | | | 134919 | | | 63637 | | | 63637 | | | 63637 | | |
|  | **Citations** | | | **Views 180** | | | **Views 2013** | | | **Altmetric** | | | **Tweets** | | | **Posts** | | |
| *Predictors* | *IRR* | *CI* | *p* | *IRR* | *CI* | *p* | *IRR* | *CI* | *p* | *b* | *CI* | *p* | *IRR* | *CI* | *p* | *IRR* | *CI* | *p* |
| (Intercept) | 5.89 | 5.66 – 6.13 | **<0.001** | 0.20 | 0.18 – 0.21 | **<0.001** | 0.16 | 0.15 – 0.16 | **<0.001** | 0.42 | 0.38 – 0.46 | **<0.001** | 2.30 | 2.12 – 2.50 | **<0.001** | 14.07 | 13.00 – 15.23 | **<0.001** |
| Promotional language | 1.10 | 1.09 – 1.11 | **<0.001** | 1.17 | 1.16 – 1.19 | **<0.001** | 1.16 | 1.15 – 1.17 | **<0.001** | 0.03 | 0.03 – 0.04 | **<0.001** | 1.03 | 1.01 – 1.04 | **<0.001** | 1.03 | 1.02 – 1.05 | **<0.001** |
| Journal [nature] | 2.96 | 2.92 – 3.00 | **<0.001** | 4.01 | 3.92 – 4.11 | **<0.001** | 4.49 | 4.42 – 4.57 | **<0.001** | 0.77 | 0.76 – 0.79 | **<0.001** | 3.33 | 3.24 – 3.43 | **<0.001** | 3.23 | 3.14 – 3.32 | **<0.001** |
| Journal [science] | 3.01 | 2.96 – 3.06 | **<0.001** | 4.59 | 4.45 – 4.73 | **<0.001** | 5.34 | 5.24 – 5.46 | **<0.001** | 0.82 | 0.80 – 0.84 | **<0.001** | 2.99 | 2.89 – 3.10 | **<0.001** | 3.28 | 3.17 – 3.40 | **<0.001** |
| Year | 1.01 | 1.01 – 1.01 | **<0.001** | 1.10 | 1.10 – 1.10 | **<0.001** | 1.12 | 1.12 – 1.12 | **<0.001** | 0.04 | 0.04 – 0.04 | **<0.001** | 1.15 | 1.14 – 1.15 | **<0.001** | 1.08 | 1.07 – 1.08 | **<0.001** |
| Word count | 1.00 | 1.00 – 1.00 | **<0.001** | 1.00 | 1.00 – 1.00 | 0.354 | 1.00 | 1.00 – 1.00 | **<0.001** | 0.00 | 0.00 – 0.00 | **<0.001** | 1.00 | 1.00 – 1.00 | **<0.001** | 1.00 | 1.00 – 1.00 | **<0.001** |
| Field [formal & natural] | 0.87 | 0.85 – 0.90 | **<0.001** | 0.99 | 0.93 – 1.04 | 0.619 | 1.08 | 1.05 – 1.13 | **<0.001** | -0.56 | -0.58 – -0.53 | **<0.001** | 0.26 | 0.25 – 0.28 | **<0.001** | 0.32 | 0.30 – 0.33 | **<0.001** |
| Field [medical & health] | 1.07 | 1.04 – 1.10 | **<0.001** | 0.57 | 0.54 – 0.61 | **<0.001** | 0.69 | 0.66 – 0.72 | **<0.001** | -0.44 | -0.47 – -0.41 | **<0.001** | 0.43 | 0.41 – 0.46 | **<0.001** | 0.52 | 0.50 – 0.55 | **<0.001** |
| Author number | 1.01 | 1.01 – 1.01 | **<0.001** | 1.00 | 1.00 – 1.00 | **<0.001** | 1.00 | 1.00 – 1.00 | **<0.001** | 0.00 | 0.00 – 0.00 | **<0.001** | 1.01 | 1.01 – 1.01 | **<0.001** | 1.01 | 1.01 – 1.01 | **<0.001** |
| Institutional diversity | 0.92 | 0.91 – 0.93 | **<0.001** | 0.99 | 0.97 – 1.01 | 0.261 | 0.99 | 0.98 – 1.01 | 0.433 | 0.07 | 0.06 – 0.08 | **<0.001** | 1.32 | 1.29 – 1.36 | **<0.001** | 1.23 | 1.20 – 1.25 | **<0.001** |
| Positivity | 1.00 | 1.00 – 1.00 | **<0.001** | 1.01 | 1.01 – 1.01 | **<0.001** | 1.01 | 1.01 – 1.01 | **<0.001** | 0.00 | 0.00 – 0.00 | **<0.001** | 1.00 | 1.00 – 1.00 | 0.118 | 1.00 | 1.00 – 1.00 | 0.918 |
| Observations | 134916 | | | 134916 | | | 134916 | | | 63634 | | | 63634 | | | 63634 | | |

*Note*. *^a^*Abstracts with a share of promotional language higher than 3 SD (n=3,300) were excluded. *b* = unstandardized regression coefficient; IRR=Incidence Rate Ratio; CI = 95% Confidence Interval. Citations: citation count per year; Views 180: the number of times the record has been accessed on the Web of Science platform (e.g. by clicking on the link to the publisher’s website or by saving it in a bibliographic management tool) in the last 180 days; Views 2013: the number of times the record has been accessed on the Web of Science platform (e.g. by clicking on the link to the publisher’s website or by saving it in a bibliographic management tool) since 2013, per year. Altmetric: Altmetric score; Tweets: number of mentions on Twitter/X; Posts: overall number of mentions in any type of online document (news, social media, policy documents etc.). Promotional language: share of promotional language in the abstract; Journal: PNAS is reference category; Year: publication year; Word count: number of words in the abstract; Field: social and humanities is reference category; author number: number of authors; institutional diversity: number of listed affiliations divided by the number of authors, higher score indicates more collaborations across different institutions; Positivity: overall semantic positivity of the abstract, obtained using the Linguistic Inquiry and Word Count software (LIWC, 2007).

Supplementary Table 5

*Effect of promotional language on public attention moderated by first author gender, without outliers^a^*

|  | **Citations** | | | **Views 180** | | | **Views 2013** | | | **Altmetric** | | | **Tweets** | | | **Posts** | | |
| --- | --- | --- | --- | --- | --- | --- | --- | --- | --- | --- | --- | --- | --- | --- | --- | --- | --- | --- |
| *Predictors* | *IRR* | *CI* | *p* | *IRR* | *CI* | *p* | *IRR* | *CI* | *p* | *b* | *CI* | *p* | *IRR* | *CI* | *p* | *IRR* | *CI* | *p* |
| (Intercept) | 15.70 | 15.41 – 15.99 | **<0.001** | 4.11 | 3.98 – 4.23 | **<0.001** | 8.31 | 8.13 – 8.50 | **<0.001** | 1.50 | 1.48 – 1.52 | **<0.001** | 88.12 | 85.17 – 91.16 | **<0.001** | 125.83 | 121.92 – 129.86 | **<0.001** |
| Promotional language | 1.11 | 1.09 – 1.12 | **<0.001** | 1.20 | 1.17 – 1.23 | **<0.001** | 1.18 | 1.16 – 1.20 | **<0.001** | 0.04 | 0.03 – 0.06 | **<0.001** | 1.08 | 1.06 – 1.11 | **<0.001** | 1.09 | 1.06 – 1.11 | **<0.001** |
| First author gender | 0.84 | 0.81 – 0.87 | **<0.001** | 0.73 | 0.70 – 0.78 | **<0.001** | 0.74 | 0.71 – 0.77 | **<0.001** | -0.09 | -0.12 – -0.06 | **<0.001** | 0.90 | 0.85 – 0.96 | **0.001** | 0.90 | 0.85 – 0.95 | **<0.001** |
| Promotional language * First author gender | 0.97 | 0.94 – 0.99 | **0.020** | 0.98 | 0.93 – 1.02 | 0.295 | 0.98 | 0.95 – 1.01 | 0.171 | 0.02 | -0.01 – 0.04 | 0.223 | 1.01 | 0.96 – 1.06 | 0.785 | 0.99 | 0.95 – 1.04 | 0.823 |
| Observations | 47519 | | | 47519 | | | 47519 | | | 34636 | | | 34636 | | | 34636 | | |
|  | **Citations** | | | **Views 180** | | | **Views 2013** | | | **Altmetric** | | | **Tweets** | | | **Posts** | | |
| *Predictors* | *IRR* | *CI* | *p* | *IRR* | *CI* | *p* | *IRR* | *CI* | *p* | *b* | *CI* | *p* | *IRR* | *CI* | *p* | *IRR* | *CI* | *p* |
| (Intercept) | 16.74 | 15.62 – 17.93 | **<0.001** | 0.19 | 0.17 – 0.21 | **<0.001** | 0.33 | 0.30 – 0.36 | **<0.001** | 0.13 | 0.06 – 0.19 | **<0.001** | 0.39 | 0.35 – 0.45 | **<0.001** | 2.36 | 2.09 – 2.67 | **<0.001** |
| Promotional language | 1.09 | 1.08 – 1.11 | **<0.001** | 1.13 | 1.11 – 1.16 | **<0.001** | 1.13 | 1.11 – 1.14 | **<0.001** | 0.04 | 0.02 – 0.05 | **<0.001** | 1.02 | 1.00 – 1.04 | 0.107 | 1.03 | 1.01 – 1.06 | **0.002** |
| First author gender | 0.93 | 0.90 – 0.95 | **<0.001** | 0.83 | 0.79 – 0.87 | **<0.001** | 0.85 | 0.82 – 0.87 | **<0.001** | -0.04 | -0.07 – -0.02 | **0.001** | 0.90 | 0.85 – 0.94 | **<0.001** | 0.87 | 0.83 – 0.92 | **<0.001** |
| Journal [nature] | 3.28 | 3.21 – 3.35 | **<0.001** | 3.58 | 3.45 – 3.72 | **<0.001** | 3.89 | 3.79 – 3.98 | **<0.001** | 0.80 | 0.78 – 0.82 | **<0.001** | 3.45 | 3.32 – 3.58 | **<0.001** | 3.54 | 3.41 – 3.67 | **<0.001** |
| Journal [science] | 3.28 | 3.19 – 3.37 | **<0.001** | 4.40 | 4.20 – 4.61 | **<0.001** | 4.76 | 4.62 – 4.91 | **<0.001** | 0.86 | 0.83 – 0.88 | **<0.001** | 3.74 | 3.56 – 3.93 | **<0.001** | 3.86 | 3.69 – 4.05 | **<0.001** |
| Year | 0.97 | 0.96 – 0.97 | **<0.001** | 1.09 | 1.09 – 1.10 | **<0.001** | 1.08 | 1.08 – 1.09 | **<0.001** | 0.05 | 0.05 – 0.06 | **<0.001** | 1.22 | 1.21 – 1.22 | **<0.001** | 1.15 | 1.15 – 1.16 | **<0.001** |
| Word count | 1.00 | 1.00 – 1.00 | **<0.001** | 1.00 | 1.00 – 1.00 | **<0.001** | 1.00 | 1.00 – 1.00 | **<0.001** | 0.00 | 0.00 – 0.00 | **<0.001** | 1.00 | 1.00 – 1.00 | **<0.001** | 1.00 | 1.00 – 1.00 | **<0.001** |
| Field [formal & natural] | 0.82 | 0.79 – 0.85 | **<0.001** | 0.84 | 0.79 – 0.90 | **<0.001** | 1.02 | 0.97 – 1.06 | 0.434 | -0.59 | -0.62 – -0.56 | **<0.001** | 0.23 | 0.22 – 0.25 | **<0.001** | 0.28 | 0.26 – 0.29 | **<0.001** |
| Field [medical & health] | 0.99 | 0.95 – 1.03 | 0.626 | 0.54 | 0.50 – 0.58 | **<0.001** | 0.67 | 0.64 – 0.71 | **<0.001** | -0.48 | -0.51 – -0.44 | **<0.001** | 0.43 | 0.40 – 0.46 | **<0.001** | 0.47 | 0.44 – 0.50 | **<0.001** |
| Author number | 1.01 | 1.01 – 1.01 | **<0.001** | 1.01 | 1.01 – 1.01 | **<0.001** | 1.01 | 1.01 – 1.01 | **<0.001** | 0.00 | 0.00 – 0.00 | **<0.001** | 1.01 | 1.01 – 1.01 | **<0.001** | 1.01 | 1.01 – 1.01 | **<0.001** |
| Institutional diversity | 0.91 | 0.90 – 0.93 | **<0.001** | 1.08 | 1.05 – 1.11 | **<0.001** | 1.07 | 1.04 – 1.09 | **<0.001** | 0.08 | 0.07 – 0.10 | **<0.001** | 1.40 | 1.35 – 1.44 | **<0.001** | 1.29 | 1.26 – 1.33 | **<0.001** |
| Positivity | 1.00 | 1.00 – 1.00 | **<0.001** | 1.01 | 1.00 – 1.01 | **<0.001** | 1.01 | 1.01 – 1.01 | **<0.001** | 0.00 | 0.00 – 0.00 | **<0.001** | 1.00 | 1.00 – 1.00 | **<0.001** | 1.00 | 1.00 – 1.00 | **<0.001** |
| Promotional language * First author gender | 0.98 | 0.96 – 1.00 | 0.060 | 0.99 | 0.96 – 1.04 | 0.787 | 0.99 | 0.96 – 1.02 | 0.504 | 0.02 | -0.00 – 0.04 | 0.092 | 0.98 | 0.94 – 1.02 | 0.360 | 1.00 | 0.96 – 1.03 | 0.806 |
| Observations | 47519 | | | 47519 | | | 47519 | | | 34636 | | | 34636 | | | 34636 | | |

*Note*. *^a^*Abstracts with a share of promotional language higher than 3 SD (n=722) were excluded. *b* = unstandardized regression coefficient; IRR=Incidence Rate Ratio; CI = 95% Confidence Interval. Citations: citation count per year; Views 180: the number of times the record has been accessed on the Web of Science platform (e.g. by clicking on the link to the publisher’s website or by saving it in a bibliographic management tool) in the last 180 days; Views 2013: the number of times the record has been accessed on the Web of Science platform (e.g. by clicking on the link to the publisher’s website or by saving it in a bibliographic management tool) since 2013, per year. Altmetric: Altmetric score; Tweets: number of mentions on Twitter/X; Posts: overall number of mentions in any type of online document (news, social media, policy documents etc.). First author gender: 1=female, 0=male. Promotional language: share of promotional language in the abstract; Journal: PNAS is reference category; Year: publication year; Word count: number of words in the abstract; Field: social and humanities is reference category; author number: number of authors; institutional diversity: number of listed affiliations divided by the number of authors, higher score indicates more collaborations across different institutions; Positivity: overall semantic positivity of the abstract, obtained using the Linguistic Inquiry and Word Count software (LIWC, 2007).

Supplementary Table 6

*Effect of promotional language on public attention moderated by last author gender, without outliers^a^*

|  | **Citations** | | | **Views 180** | | | **Views 2013** | | | **Altmetric** | | | **Tweets** | | | **Posts** | | |
| --- | --- | --- | --- | --- | --- | --- | --- | --- | --- | --- | --- | --- | --- | --- | --- | --- | --- | --- |
| *Predictors* | *IRR* | *CI* | *p* | *IRR* | *CI* | *p* | *IRR* | *CI* | *p* | *b* | *CI* | *p* | *IRR* | *CI* | *p* | *IRR* | *CI* | *p* |
| (Intercept) | 15.20 | 14.97 – 15.44 | **<0.001** | 4.01 | 3.91 – 4.12 | **<0.001** | 8.19 | 8.04 – 8.34 | **<0.001** | 1.44 | 1.42 – 1.45 | **<0.001** | 81.42 | 79.15 – 83.76 | **<0.001** | 116.10 | 113.08 – 119.20 | **<0.001** |
| Promotional language | 1.11 | 1.09 – 1.12 | **<0.001** | 1.22 | 1.19 – 1.25 | **<0.001** | 1.19 | 1.18 – 1.21 | **<0.001** | 0.06 | 0.05 – 0.07 | **<0.001** | 1.09 | 1.07 – 1.12 | **<0.001** | 1.10 | 1.07 – 1.12 | **<0.001** |
| Last author gender | 0.88 | 0.85 – 0.91 | **<0.001** | 0.94 | 0.88 – 1.00 | **0.045** | 0.88 | 0.84 – 0.92 | **<0.001** | 0.04 | 0.00 – 0.07 | **0.024** | 1.03 | 0.96 – 1.10 | 0.462 | 1.01 | 0.95 – 1.07 | 0.781 |
| Promotional language * Last author gender | 0.95 | 0.92 – 0.98 | **<0.001** | 0.92 | 0.87 – 0.97 | **0.001** | 0.95 | 0.91 – 0.98 | **0.004** | -0.05 | -0.08 – -0.02 | **0.001** | 0.93 | 0.88 – 0.99 | **0.016** | 0.94 | 0.89 – 0.99 | **0.017** |
| Observations | 55998 | | | 55998 | | | 55998 | | | 40802 | | | 40802 | | | 40802 | | |
|  | **Citations** | | | **Views 180** | | | **Views 2013** | | | **Altmetric** | | | **Tweets** | | | **Posts** | | |
| *Predictors* | *IRR* | *CI* | *p* | *IRR* | *CI* | *p* | *IRR* | *CI* | *p* | *b* | *CI* | *p* | *IRR* | *CI* | *p* | *IRR* | *CI* | *p* |
| (Intercept) | 16.42 | 15.39 – 17.51 | **<0.001** | 0.16 | 0.14 – 0.18 | **<0.001** | 0.28 | 0.26 – 0.31 | **<0.001** | 0.15 | 0.09 – 0.21 | **<0.001** | 0.25 | 0.22 – 0.28 | **<0.001** | 1.76 | 1.57 – 1.97 | **<0.001** |
| Promotional language | 1.10 | 1.09 – 1.11 | **<0.001** | 1.15 | 1.13 – 1.17 | **<0.001** | 1.14 | 1.12 – 1.15 | **<0.001** | 0.05 | 0.04 – 0.06 | **<0.001** | 1.03 | 1.01 – 1.05 | **0.006** | 1.05 | 1.03 – 1.07 | **<0.001** |
| Last author gender | 0.97 | 0.94 – 1.00 | **0.039** | 0.95 | 0.90 – 1.00 | 0.061 | 0.93 | 0.90 – 0.97 | **<0.001** | 0.04 | 0.02 – 0.07 | **0.002** | 0.85 | 0.80 – 0.90 | **<0.001** | 0.89 | 0.85 – 0.94 | **<0.001** |
| Journal [nature] | 3.30 | 3.24 – 3.37 | **<0.001** | 3.67 | 3.55 – 3.80 | **<0.001** | 4.07 | 3.97 – 4.16 | **<0.001** | 0.81 | 0.79 – 0.83 | **<0.001** | 3.56 | 3.43 – 3.69 | **<0.001** | 3.57 | 3.45 – 3.69 | **<0.001** |
| Journal [science] | 3.43 | 3.34 – 3.52 | **<0.001** | 4.74 | 4.54 – 4.95 | **<0.001** | 5.21 | 5.06 – 5.36 | **<0.001** | 0.87 | 0.84 – 0.89 | **<0.001** | 4.16 | 3.98 – 4.35 | **<0.001** | 4.11 | 3.94 – 4.29 | **<0.001** |
| Year | 0.96 | 0.96 – 0.97 | **<0.001** | 1.09 | 1.09 – 1.10 | **<0.001** | 1.09 | 1.08 – 1.09 | **<0.001** | 0.05 | 0.05 – 0.05 | **<0.001** | 1.23 | 1.22 – 1.23 | **<0.001** | 1.16 | 1.15 – 1.16 | **<0.001** |
| Word count | 1.00 | 1.00 – 1.00 | **<0.001** | 1.00 | 1.00 – 1.00 | **<0.001** | 1.00 | 1.00 – 1.00 | **<0.001** | 0.00 | 0.00 – 0.00 | **<0.001** | 1.00 | 1.00 – 1.00 | **<0.001** | 1.00 | 1.00 – 1.00 | **<0.001** |
| Field [formal & natural] | 0.82 | 0.79 – 0.85 | **<0.001** | 0.86 | 0.81 – 0.92 | **<0.001** | 1.04 | 1.00 – 1.09 | 0.056 | -0.61 | -0.64 – -0.58 | **<0.001** | 0.22 | 0.21 – 0.24 | **<0.001** | 0.27 | 0.25 – 0.28 | **<0.001** |
| Field [medical & health] | 0.96 | 0.92 – 1.00 | 0.066 | 0.52 | 0.49 – 0.56 | **<0.001** | 0.66 | 0.63 – 0.69 | **<0.001** | -0.50 | -0.54 – -0.47 | **<0.001** | 0.39 | 0.37 – 0.42 | **<0.001** | 0.44 | 0.41 – 0.47 | **<0.001** |
| Author number | 1.02 | 1.02 – 1.02 | **<0.001** | 1.01 | 1.01 – 1.01 | **<0.001** | 1.01 | 1.01 – 1.01 | **<0.001** | 0.00 | 0.00 – 0.00 | **<0.001** | 1.01 | 1.01 – 1.01 | **<0.001** | 1.01 | 1.01 – 1.01 | **<0.001** |
| Institutional diversity | 0.94 | 0.92 – 0.95 | **<0.001** | 1.08 | 1.05 – 1.11 | **<0.001** | 1.06 | 1.04 – 1.08 | **<0.001** | 0.09 | 0.07 – 0.10 | **<0.001** | 1.52 | 1.47 – 1.56 | **<0.001** | 1.39 | 1.35 – 1.43 | **<0.001** |
| Positivity | 1.00 | 1.00 – 1.00 | **<0.001** | 1.01 | 1.01 – 1.01 | **<0.001** | 1.01 | 1.01 – 1.01 | **<0.001** | 0.00 | 0.00 – 0.00 | **<0.001** | 1.00 | 1.00 – 1.00 | **<0.001** | 1.00 | 1.00 – 1.00 | **0.004** |
| Promotional language * Last author gender | 0.97 | 0.95 – 1.00 | **0.030** | 1.00 | 0.96 – 1.04 | 0.966 | 1.01 | 0.98 – 1.04 | 0.697 | -0.03 | -0.05 – -0.00 | **0.030** | 1.01 | 0.97 – 1.06 | 0.572 | 1.00 | 0.96 – 1.04 | 0.940 |
| Observations | 55998 | | | 55998 | | | 55998 | | | 40802 | | | 40802 | | | 40802 | | |

*Note*. *^a^*Abstracts with a share of promotional language higher than 3 SD (n=826) were excluded. *b* = unstandardized regression coefficient; IRR=Incidence Rate Ratio; CI = 95% Confidence Interval. Citations: citation count per year; Views 180: the number of times the record has been accessed on the Web of Science platform (e.g. by clicking on the link to the publisher’s website or by saving it in a bibliographic management tool) in the last 180 days; Views 2013: the number of times the record has been accessed on the Web of Science platform (e.g. by clicking on the link to the publisher’s website or by saving it in a bibliographic management tool) since 2013, per year. Altmetric: Altmetric score; Tweets: number of mentions on Twitter/X; Posts: overall number of mentions in any type of online document (news, social media, policy documents etc.). Last author gender: 1=female, 0=male. Promotional language: share of promotional language in the abstract; Journal: PNAS is reference category; Year: publication year; Word count: number of words in the abstract; Field: social and humanities is reference category; author number: number of authors; institutional diversity: number of listed affiliations divided by the number of authors, higher score indicates more collaborations across different institutions; Positivity: overall semantic positivity of the abstract, obtained using the Linguistic Inquiry and Word Count software (LIWC, 2007).

Supplementary Table 7

*Promotional language, academic impact and public attention, with papers published in 2023 excluded*

|  | **Citations** | | | **Views 180** | | | **Views 2013** | | | **Altmetric** | | | **Tweets** | | | **Posts** | | |
| --- | --- | --- | --- | --- | --- | --- | --- | --- | --- | --- | --- | --- | --- | --- | --- | --- | --- | --- |
| *Predictors* | *IRR* | *CI* | *p* | *IRR* | *CI* | *p* | *IRR* | *CI* | *p* | *b* | *CI* | *p* | *IRR* | *CI* | *p* | *IRR* | *CI* | *p* |
| (Intercept) | 13.28 | 13.17 – 13.39 | **<0.001** | 2.81 | 2.76 – 2.85 | **<0.001** | 5.20 | 5.14 – 5.26 | **<0.001** | 1.37 | 1.36 – 1.38 | **<0.001** | 65.10 | 63.81 – 66.41 | **<0.001** | 97.75 | 96.00 – 99.54 | **<0.001** |
| Promotional language | 1.14 | 1.14 – 1.15 | **<0.001** | 1.37 | 1.35 – 1.39 | **<0.001** | 1.37 | 1.35 – 1.38 | **<0.001** | 0.06 | 0.05 – 0.06 | **<0.001** | 1.12 | 1.10 – 1.13 | **<0.001** | 1.11 | 1.09 – 1.12 | **<0.001** |
| Observations | 136198 | | | 136198 | | | 136198 | | | 64290 | | | 64290 | | | 64290 | | |
|  | **Citations** | | | **Views 180** | | | **Views 2013** | | | **Altmetric** | | | **Tweets** | | | **Posts** | | |
| *Predictors* | *IRR* | *CI* | *p* | *IRR* | *CI* | *p* | *IRR* | *CI* | *p* | *b* | *CI* | *p* | *IRR* | *CI* | *p* | *IRR* | *CI* | *p* |
| (Intercept) | 5.81 | 5.58 – 6.04 | **<0.001** | 0.20 | 0.18 – 0.21 | **<0.001** | 0.15 | 0.15 – 0.16 | **<0.001** | 0.42 | 0.38 – 0.46 | **<0.001** | 2.25 | 2.07 – 2.44 | **<0.001** | 13.89 | 12.84 – 15.02 | **<0.001** |
| Promotional language | 1.09 | 1.08 – 1.10 | **<0.001** | 1.16 | 1.14 – 1.17 | **<0.001** | 1.14 | 1.13 – 1.15 | **<0.001** | 0.03 | 0.03 – 0.04 | **<0.001** | 1.04 | 1.03 – 1.05 | **<0.001** | 1.05 | 1.03 – 1.06 | **<0.001** |
| Journal [nature] | 2.96 | 2.92 – 3.00 | **<0.001** | 3.99 | 3.90 – 4.09 | **<0.001** | 4.50 | 4.42 – 4.57 | **<0.001** | 0.77 | 0.76 – 0.78 | **<0.001** | 3.32 | 3.22 – 3.41 | **<0.001** | 3.20 | 3.12 – 3.29 | **<0.001** |
| Journal [science] | 3.01 | 2.96 – 3.06 | **<0.001** | 4.56 | 4.43 – 4.70 | **<0.001** | 5.33 | 5.22 – 5.44 | **<0.001** | 0.82 | 0.80 – 0.84 | **<0.001** | 2.97 | 2.87 – 3.08 | **<0.001** | 3.25 | 3.14 – 3.36 | **<0.001** |
| Year | 1.01 | 1.01 – 1.01 | **<0.001** | 1.10 | 1.10 – 1.10 | **<0.001** | 1.12 | 1.12 – 1.12 | **<0.001** | 0.04 | 0.04 – 0.04 | **<0.001** | 1.15 | 1.15 – 1.15 | **<0.001** | 1.08 | 1.07 – 1.08 | **<0.001** |
| Word count | 1.00 | 1.00 – 1.00 | **<0.001** | 1.00 | 1.00 – 1.00 | 0.480 | 1.00 | 1.00 – 1.00 | **<0.001** | 0.00 | 0.00 – 0.00 | **<0.001** | 1.00 | 1.00 – 1.00 | **<0.001** | 1.00 | 1.00 – 1.00 | **<0.001** |
| Field [formal & natural] | 0.88 | 0.85 – 0.91 | **<0.001** | 0.99 | 0.94 – 1.04 | 0.702 | 1.09 | 1.05 – 1.13 | **<0.001** | -0.55 | -0.58 – -0.53 | **<0.001** | 0.27 | 0.25 – 0.28 | **<0.001** | 0.32 | 0.30 – 0.33 | **<0.001** |
| Field [medical & health] | 1.08 | 1.04 – 1.11 | **<0.001** | 0.57 | 0.54 – 0.61 | **<0.001** | 0.69 | 0.66 – 0.72 | **<0.001** | -0.44 | -0.46 – -0.41 | **<0.001** | 0.44 | 0.42 – 0.47 | **<0.001** | 0.53 | 0.50 – 0.56 | **<0.001** |
| Author number | 1.01 | 1.01 – 1.01 | **<0.001** | 1.00 | 1.00 – 1.00 | **<0.001** | 1.00 | 1.00 – 1.00 | **<0.001** | 0.00 | 0.00 – 0.00 | **<0.001** | 1.01 | 1.01 – 1.01 | **<0.001** | 1.01 | 1.01 – 1.01 | **<0.001** |
| Institutional diversity | 0.92 | 0.91 – 0.94 | **<0.001** | 0.99 | 0.97 – 1.01 | 0.465 | 1.00 | 0.98 – 1.01 | 0.655 | 0.07 | 0.06 – 0.08 | **<0.001** | 1.32 | 1.29 – 1.35 | **<0.001** | 1.22 | 1.19 – 1.25 | **<0.001** |
| Positivity | 1.00 | 1.00 – 1.00 | **<0.001** | 1.01 | 1.01 – 1.01 | **<0.001** | 1.01 | 1.01 – 1.01 | **<0.001** | 0.00 | 0.00 – 0.00 | **<0.001** | 1.00 | 1.00 – 1.00 | 0.671 | 1.00 | 1.00 – 1.00 | 0.346 |
| Observations | 136195 | | | 136195 | | | 136195 | | | 64287 | | | 64287 | | | 64287 | | |

*Note*. *b* = unstandardized regression coefficient; IRR=Incidence Rate Ratio; CI = 95% Confidence Interval. Citations: citation count per year; Views 180: the number of times the record has been accessed on the Web of Science platform (e.g. by clicking on the link to the publisher’s website or by saving it in a bibliographic management tool) in the last 180 days; Views 2013: the number of times the record has been accessed on the Web of Science platform (e.g. by clicking on the link to the publisher’s website or by saving it in a bibliographic management tool) since 2013, per year. Altmetric: Altmetric score; Tweets: number of mentions on Twitter/X; Posts: overall number of mentions in any type of online document (news, social media, policy documents etc.). Promotional language: share of promotional language in the abstract; Journal: PNAS is reference category; Year: publication year; Word count: number of words in the abstract; Field: social and humanities is reference category; author number: number of authors; institutional diversity: number of listed affiliations divided by the number of authors, higher score indicates more collaborations across different institutions; Positivity: overall semantic positivity of the abstract, obtained using the Linguistic Inquiry and Word Count software (LIWC, 2007).

Supplementary Table 8

*Effect of promotional language on public attention moderated by first author gender, with papers published in 2023 excluded*

|  | **Citations** | | | **Views 180** | | | **Views 2013** | | | **Altmetric** | | | **Tweets** | | | **Posts** | | |
| --- | --- | --- | --- | --- | --- | --- | --- | --- | --- | --- | --- | --- | --- | --- | --- | --- | --- | --- |
| *Predictors* | *IRR* | *CI* | *p* | *IRR* | *CI* | *p* | *IRR* | *CI* | *p* | *b* | *CI* | *p* | *IRR* | *CI* | *p* | *IRR* | *CI* | *p* |
| (Intercept) | 15.86 | 15.59 – 16.15 | **<0.001** | 4.11 | 3.99 – 4.23 | **<0.001** | 8.41 | 8.24 – 8.59 | **<0.001** | 1.49 | 1.48 – 1.51 | **<0.001** | 86.71 | 83.94 – 89.57 | **<0.001** | 124.11 | 120.42 – 127.91 | **<0.001** |
| Promotional language | 1.10 | 1.08 – 1.11 | **<0.001** | 1.18 | 1.15 – 1.21 | **<0.001** | 1.16 | 1.14 – 1.18 | **<0.001** | 0.05 | 0.04 – 0.06 | **<0.001** | 1.09 | 1.06 – 1.11 | **<0.001** | 1.09 | 1.07 – 1.11 | **<0.001** |
| First author gender | 0.83 | 0.81 – 0.86 | **<0.001** | 0.72 | 0.68 – 0.76 | **<0.001** | 0.72 | 0.70 – 0.75 | **<0.001** | -0.08 | -0.11 – -0.05 | **<0.001** | 0.93 | 0.88 – 0.98 | **0.011** | 0.92 | 0.87 – 0.97 | **0.002** |
| Promotional language * First author gender | 0.98 | 0.95 – 1.00 | 0.057 | 1.00 | 0.96 – 1.04 | 0.990 | 1.00 | 0.97 – 1.03 | 0.875 | 0.01 | -0.02 – 0.03 | 0.611 | 0.98 | 0.93 – 1.02 | 0.290 | 0.97 | 0.93 – 1.01 | 0.121 |
| Observations | 47519 | | | 47519 | | | 47519 | | | 34636 | | | 34636 | | | 34636 | | |
|  | **Citations** | | | **Views 180** | | | **Views 2013** | | | **Altmetric** | | | **Tweets** | | | **Posts** | | |
| *Predictors* | *IRR* | *CI* | *p* | *IRR* | *CI* | *p* | *IRR* | *CI* | *p* | *b* | *CI* | *p* | *IRR* | *CI* | *p* | *IRR* | *CI* | *p* |
| (Intercept) | 15.95 | 14.90 – 17.07 | **<0.001** | 0.19 | 0.17 – 0.21 | **<0.001** | 0.32 | 0.30 – 0.35 | **<0.001** | 0.11 | 0.05 – 0.18 | **0.001** | 0.37 | 0.33 – 0.42 | **<0.001** | 2.27 | 2.01 – 2.57 | **<0.001** |
| Promotional language | 1.08 | 1.07 – 1.10 | **<0.001** | 1.12 | 1.10 – 1.14 | **<0.001** | 1.11 | 1.09 – 1.12 | **<0.001** | 0.04 | 0.03 – 0.05 | **<0.001** | 1.05 | 1.03 – 1.07 | **<0.001** | 1.06 | 1.04 – 1.08 | **<0.001** |
| First author gender | 0.93 | 0.90 – 0.95 | **<0.001** | 0.82 | 0.78 – 0.86 | **<0.001** | 0.84 | 0.81 – 0.86 | **<0.001** | -0.04 | -0.06 – -0.01 | **0.002** | 0.93 | 0.88 – 0.97 | **0.002** | 0.90 | 0.86 – 0.94 | **<0.001** |
| Journal [nature] | 3.28 | 3.21 – 3.35 | **<0.001** | 3.55 | 3.42 – 3.68 | **<0.001** | 3.88 | 3.78 – 3.97 | **<0.001** | 0.80 | 0.78 – 0.82 | **<0.001** | 3.44 | 3.31 – 3.57 | **<0.001** | 3.52 | 3.40 – 3.66 | **<0.001** |
| Journal [science] | 3.28 | 3.20 – 3.37 | **<0.001** | 4.38 | 4.18 – 4.58 | **<0.001** | 4.76 | 4.61 – 4.91 | **<0.001** | 0.85 | 0.83 – 0.88 | **<0.001** | 3.71 | 3.54 – 3.90 | **<0.001** | 3.83 | 3.66 – 4.01 | **<0.001** |
| Year | 0.97 | 0.97 – 0.97 | **<0.001** | 1.09 | 1.09 – 1.10 | **<0.001** | 1.08 | 1.08 – 1.09 | **<0.001** | 0.05 | 0.05 – 0.06 | **<0.001** | 1.22 | 1.22 – 1.22 | **<0.001** | 1.16 | 1.15 – 1.16 | **<0.001** |
| Word count | 1.00 | 1.00 – 1.00 | **<0.001** | 1.00 | 1.00 – 1.00 | **<0.001** | 1.00 | 1.00 – 1.00 | **<0.001** | 0.00 | 0.00 – 0.00 | **<0.001** | 1.00 | 1.00 – 1.00 | **<0.001** | 1.00 | 1.00 – 1.00 | **<0.001** |
| Field [formal & natural] | 0.82 | 0.79 – 0.86 | **<0.001** | 0.85 | 0.79 – 0.90 | **<0.001** | 1.02 | 0.98 – 1.07 | 0.304 | -0.59 | -0.62 – -0.56 | **<0.001** | 0.23 | 0.22 – 0.24 | **<0.001** | 0.28 | 0.26 – 0.29 | **<0.001** |
| Field [medical & health] | 0.99 | 0.95 – 1.04 | 0.793 | 0.54 | 0.51 – 0.58 | **<0.001** | 0.68 | 0.64 – 0.71 | **<0.001** | -0.47 | -0.51 – -0.44 | **<0.001** | 0.43 | 0.40 – 0.46 | **<0.001** | 0.47 | 0.44 – 0.50 | **<0.001** |
| Author number | 1.01 | 1.01 – 1.01 | **<0.001** | 1.01 | 1.01 – 1.01 | **<0.001** | 1.01 | 1.01 – 1.01 | **<0.001** | 0.00 | 0.00 – 0.00 | **<0.001** | 1.01 | 1.01 – 1.01 | **<0.001** | 1.01 | 1.01 – 1.01 | **<0.001** |
| Institutional diversity | 0.91 | 0.90 – 0.93 | **<0.001** | 1.08 | 1.05 – 1.11 | **<0.001** | 1.07 | 1.04 – 1.09 | **<0.001** | 0.08 | 0.07 – 0.10 | **<0.001** | 1.39 | 1.35 – 1.43 | **<0.001** | 1.28 | 1.25 – 1.32 | **<0.001** |
| Positivity | 1.00 | 1.00 – 1.00 | **<0.001** | 1.01 | 1.00 – 1.01 | **<0.001** | 1.01 | 1.01 – 1.01 | **<0.001** | 0.00 | 0.00 – 0.00 | **<0.001** | 1.00 | 1.00 – 1.00 | **<0.001** | 1.00 | 1.00 – 1.00 | **<0.001** |
| Promotional language * First author gender | 0.98 | 0.96 – 1.00 | **0.023** | 1.01 | 0.97 – 1.04 | 0.691 | 1.00 | 0.98 – 1.03 | 0.854 | 0.01 | -0.01 – 0.03 | 0.404 | 0.94 | 0.91 – 0.98 | **0.002** | 0.95 | 0.92 – 0.99 | **0.008** |
| Observations | 47519 | | | 47519 | | | 47519 | | | 34636 | | | 34636 | | | 34636 | | |

*Note*. *b* = unstandardized regression coefficient; IRR=Incidence Rate Ratio; CI = 95% Confidence Interval. Citations: citation count per year; Views 180: the number of times the record has been accessed on the Web of Science platform (e.g. by clicking on the link to the publisher’s website or by saving it in a bibliographic management tool) in the last 180 days; Views 2013: the number of times the record has been accessed on the Web of Science platform (e.g. by clicking on the link to the publisher’s website or by saving it in a bibliographic management tool) since 2013, per year. Altmetric: Altmetric score; Tweets: number of mentions on Twitter/X; Posts: overall number of mentions in any type of online document (news, social media, policy documents etc.). First author gender: 1=female, 0=male. Promotional language: share of promotional language in the abstract; Journal: PNAS is reference category; Year: publication year; Word count: number of words in the abstract; Field: social and humanities is reference category; author number: number of authors; institutional diversity: number of listed affiliations divided by the number of authors, higher score indicates more collaborations across different institutions; Positivity: overall semantic positivity of the abstract, obtained using the Linguistic Inquiry and Word Count software (LIWC, 2007).

Supplementary Table 9

*Effect of promotional language on public attention moderated by last author gender, with papers published in 2023 excluded*

|  | **Citations** | | | **Views 180** | | | **Views 2013** | | | **Altmetric** | | | **Tweets** | | | **Posts** | | |
| --- | --- | --- | --- | --- | --- | --- | --- | --- | --- | --- | --- | --- | --- | --- | --- | --- | --- | --- |
| *Predictors* | *IRR* | *CI* | *p* | *IRR* | *CI* | *p* | *IRR* | *CI* | *p* | *b* | *CI* | *p* | *IRR* | *CI* | *p* | *IRR* | *CI* | *p* |
| (Intercept) | 15.33 | 15.11 – 15.56 | **<0.001** | 4.00 | 3.90 – 4.10 | **<0.001** | 8.25 | 8.10 – 8.40 | **<0.001** | 1.44 | 1.42 – 1.45 | **<0.001** | 80.81 | 78.64 – 83.04 | **<0.001** | 115.33 | 112.44 – 118.29 | **<0.001** |
| Promotional language | 1.10 | 1.09 – 1.11 | **<0.001** | 1.20 | 1.18 – 1.23 | **<0.001** | 1.18 | 1.16 – 1.19 | **<0.001** | 0.06 | 0.05 – 0.07 | **<0.001** | 1.09 | 1.07 – 1.11 | **<0.001** | 1.09 | 1.07 – 1.11 | **<0.001** |
| Last author gender | 0.88 | 0.85 – 0.91 | **<0.001** | 0.92 | 0.87 – 0.98 | **0.010** | 0.86 | 0.83 – 0.90 | **<0.001** | 0.03 | -0.00 – 0.06 | 0.060 | 1.00 | 0.94 – 1.06 | 0.949 | 0.99 | 0.93 – 1.05 | 0.719 |
| Promotional language * Last author gender | 0.95 | 0.92 – 0.98 | **<0.001** | 0.94 | 0.89 – 0.98 | **0.005** | 0.96 | 0.93 – 1.00 | **0.032** | -0.04 | -0.06 – -0.01 | **0.004** | 0.96 | 0.91 – 1.01 | 0.118 | 0.96 | 0.92 – 1.00 | 0.076 |
| Observations | 55998 | | | 55998 | | | 55998 | | | 40802 | | | 40802 | | | 40802 | | |
|  | **Citations** | | | **Views 180** | | | **Views 2013** | | | **Altmetric** | | | **Tweets** | | | **Posts** | | |
| *Predictors* | *IRR* | *CI* | *p* | *IRR* | *CI* | *p* | *IRR* | *CI* | *p* | *b* | *CI* | *p* | *IRR* | *CI* | *p* | *IRR* | *CI* | *p* |
| (Intercept) | 15.58 | 14.62 – 16.60 | **<0.001** | 0.16 | 0.14 – 0.18 | **<0.001** | 0.28 | 0.26 – 0.30 | **<0.001** | 0.14 | 0.08 – 0.20 | **<0.001** | 0.23 | 0.21 – 0.26 | **<0.001** | 1.70 | 1.52 – 1.91 | **<0.001** |
| Promotional language | 1.09 | 1.08 – 1.10 | **<0.001** | 1.14 | 1.12 – 1.16 | **<0.001** | 1.12 | 1.11 – 1.13 | **<0.001** | 0.05 | 0.04 – 0.06 | **<0.001** | 1.05 | 1.03 – 1.07 | **<0.001** | 1.07 | 1.05 – 1.08 | **<0.001** |
| Last author gender | 0.97 | 0.94 – 1.00 | 0.057 | 0.96 | 0.91 – 1.01 | 0.091 | 0.93 | 0.90 – 0.97 | **<0.001** | 0.04 | 0.02 – 0.07 | **0.001** | 0.86 | 0.82 – 0.91 | **<0.001** | 0.91 | 0.87 – 0.96 | **<0.001** |
| Journal [nature] | 3.31 | 3.24 – 3.38 | **<0.001** | 3.64 | 3.52 – 3.77 | **<0.001** | 4.06 | 3.97 – 4.16 | **<0.001** | 0.81 | 0.79 – 0.82 | **<0.001** | 3.54 | 3.42 – 3.67 | **<0.001** | 3.55 | 3.43 – 3.67 | **<0.001** |
| Journal [science] | 3.45 | 3.36 – 3.53 | **<0.001** | 4.73 | 4.53 – 4.94 | **<0.001** | 5.21 | 5.06 – 5.36 | **<0.001** | 0.86 | 0.84 – 0.89 | **<0.001** | 4.13 | 3.95 – 4.32 | **<0.001** | 4.07 | 3.90 – 4.24 | **<0.001** |
| Year | 0.97 | 0.96 – 0.97 | **<0.001** | 1.09 | 1.09 – 1.10 | **<0.001** | 1.09 | 1.08 – 1.09 | **<0.001** | 0.05 | 0.05 – 0.05 | **<0.001** | 1.23 | 1.23 – 1.23 | **<0.001** | 1.16 | 1.16 – 1.16 | **<0.001** |
| Word count | 1.00 | 1.00 – 1.00 | **<0.001** | 1.00 | 1.00 – 1.00 | **<0.001** | 1.00 | 1.00 – 1.00 | **<0.001** | 0.00 | 0.00 – 0.00 | **<0.001** | 1.00 | 1.00 – 1.00 | **<0.001** | 1.00 | 1.00 – 1.00 | **<0.001** |
| Field [formal & natural] | 0.82 | 0.79 – 0.85 | **<0.001** | 0.87 | 0.82 – 0.92 | **<0.001** | 1.05 | 1.00 – 1.09 | **0.029** | -0.60 | -0.63 – -0.57 | **<0.001** | 0.22 | 0.21 – 0.24 | **<0.001** | 0.27 | 0.25 – 0.28 | **<0.001** |
| Field [medical & health] | 0.97 | 0.93 – 1.01 | 0.095 | 0.53 | 0.49 – 0.56 | **<0.001** | 0.66 | 0.63 – 0.69 | **<0.001** | -0.50 | -0.53 – -0.46 | **<0.001** | 0.39 | 0.37 – 0.42 | **<0.001** | 0.44 | 0.41 – 0.47 | **<0.001** |
| Author number | 1.02 | 1.02 – 1.02 | **<0.001** | 1.01 | 1.01 – 1.01 | **<0.001** | 1.01 | 1.01 – 1.01 | **<0.001** | 0.00 | 0.00 – 0.00 | **<0.001** | 1.01 | 1.01 – 1.01 | **<0.001** | 1.01 | 1.01 – 1.01 | **<0.001** |
| Institutional diversity | 0.94 | 0.92 – 0.95 | **<0.001** | 1.08 | 1.05 – 1.11 | **<0.001** | 1.06 | 1.04 – 1.08 | **<0.001** | 0.08 | 0.07 – 0.10 | **<0.001** | 1.51 | 1.47 – 1.55 | **<0.001** | 1.38 | 1.34 – 1.41 | **<0.001** |
| Positivity | 1.00 | 1.00 – 1.00 | **<0.001** | 1.01 | 1.01 – 1.01 | **<0.001** | 1.01 | 1.01 – 1.01 | **<0.001** | 0.00 | 0.00 – 0.00 | **<0.001** | 1.00 | 1.00 – 1.00 | **<0.001** | 1.00 | 1.00 – 1.00 | **<0.001** |
| Promotional language * Last author gender | 0.97 | 0.94 – 0.99 | **0.004** | 0.99 | 0.95 – 1.03 | 0.507 | 1.00 | 0.98 – 1.03 | 0.824 | -0.03 | -0.05 – -0.00 | **0.015** | 0.99 | 0.95 – 1.03 | 0.621 | 0.97 | 0.93 – 1.01 | 0.133 |
| Observations | 55998 | | | 55998 | | | 55998 | | | 40802 | | | 40802 | | | 40802 | | |

*Note*. *b* = unstandardized regression coefficient; IRR=Incidence Rate Ratio; CI = 95% Confidence Interval. Citations: citation count per year; Views 180: the number of times the record has been accessed on the Web of Science platform (e.g. by clicking on the link to the publisher’s website or by saving it in a bibliographic management tool) in the last 180 days; Views 2013: the number of times the record has been accessed on the Web of Science platform (e.g. by clicking on the link to the publisher’s website or by saving it in a bibliographic management tool) since 2013, per year. Altmetric: Altmetric score; Tweets: number of mentions on Twitter/X; Posts: overall number of mentions in any type of online document (news, social media, policy documents etc.). Last author gender: 1=female, 0=male. Promotional language: share of promotional language in the abstract; Journal: PNAS is reference category; Year: publication year; Word count: number of words in the abstract; Field: social and humanities is reference category; author number: number of authors; institutional diversity: number of listed affiliations divided by the number of authors, higher score indicates more collaborations across different institutions; Positivity: overall semantic positivity of the abstract, obtained using the Linguistic Inquiry and Word Count software (LIWC, 2007).

# Promotional language and impact over time

Supplementary Table 10

*Effect of promotional language on academic impact and public attention across time*

|  | **Citations** | | | **Views 180** | | | **Views 2013** | | | **Altmetric** | | | **Tweets** | | | **Posts** | | |
| --- | --- | --- | --- | --- | --- | --- | --- | --- | --- | --- | --- | --- | --- | --- | --- | --- | --- | --- |
| *Predictors* | *IRR* | *CI* | *p* | *IRR* | *CI* | *p* | *IRR* | *CI* | *p* | *b* | *CI* | *p* | *IRR* | *CI* | *p* | *IRR* | *CI* | *p* |
| (Intercept) | 9.748 | 9.594 – 9.905 | **<0.001** | 0.511 | 0.496 – 0.527 | **<0.001** | 0.569 | 0.557 – 0.583 | **<0.001** | 0.692 | 0.662 – 0.722 | **<0.001** | 1.806 | 1.702 – 1.916 | **<0.001** | 14.382 | 13.608 – 15.201 | **<0.001** |
| Promotional language | 1.185 | 1.167 – 1.203 | **<0.001** | 1.258 | 1.223 – 1.294 | **<0.001** | 1.310 | 1.283 – 1.337 | **<0.001** | -0.001 | -0.026 – 0.024 | 0.930 | 0.944 | 0.899 – 0.992 | **0.022** | 0.927 | 0.886 – 0.971 | **0.001** |
| Year | 1.020 | 1.019 – 1.021 | **<0.001** | 1.097 | 1.096 – 1.099 | **<0.001** | 1.124 | 1.123 – 1.126 | **<0.001** | 0.031 | 0.030 – 0.032 | **<0.001** | 1.152 | 1.149 – 1.155 | **<0.001** | 1.082 | 1.080 – 1.085 | **<0.001** |
| Promotional language * Year | 0.996 | 0.995 – 0.997 | **<0.001** | 0.997 | 0.996 – 0.999 | **<0.001** | 0.994 | 0.993 – 0.995 | **<0.001** | 0.002 | 0.001 – 0.003 | **0.004** | 1.005 | 1.003 – 1.007 | **<0.001** | 1.005 | 1.004 – 1.007 | **<0.001** |
| Observations | 136615 | | | 136615 | | | 136615 | | | 64693 | | | 64693 | | | 64693 | | |
|  | **Citations** | | | **Views 180** | | | **Views 2013** | | | **Altmetric** | | | **Tweets** | | | **Posts** | | |
| *Predictors* | *IRR* | *CI* | *p* | *IRR* | *CI* | *p* | *IRR* | *CI* | *p* | *b* | *CI* | *p* | *IRR* | *CI* | *p* | *IRR* | *CI* | *p* |
| (Intercept) | 5.584 | 5.362 – 5.816 | **<0.001** | 0.195 | 0.181 – 0.210 | **<0.001** | 0.147 | 0.139 – 0.155 | **<0.001** | 0.445 | 0.401 – 0.489 | **<0.001** | 2.516 | 2.298 – 2.756 | **<0.001** | 15.669 | 14.383 – 17.069 | **<0.001** |
| Promotional language | 1.167 | 1.151 – 1.183 | **<0.001** | 1.172 | 1.143 – 1.203 | **<0.001** | 1.232 | 1.210 – 1.255 | **<0.001** | 0.001 | -0.021 – 0.023 | 0.922 | 0.923 | 0.881 – 0.966 | **0.001** | 0.912 | 0.874 – 0.952 | **<0.001** |
| Journal [nature] | 1.015 | 1.015 – 1.016 | **<0.001** | 1.100 | 1.098 – 1.102 | **<0.001** | 1.126 | 1.125 – 1.127 | **<0.001** | 0.038 | 0.037 – 0.039 | **<0.001** | 1.142 | 1.139 – 1.145 | **<0.001** | 1.070 | 1.068 – 1.073 | **<0.001** |
| Journal [science] | 2.946 | 2.908 – 2.985 | **<0.001** | 4.002 | 3.909 – 4.098 | **<0.001** | 4.478 | 4.406 – 4.552 | **<0.001** | 0.771 | 0.757 – 0.785 | **<0.001** | 3.323 | 3.231 – 3.417 | **<0.001** | 3.219 | 3.135 – 3.307 | **<0.001** |
| Year | 3.003 | 2.955 – 3.052 | **<0.001** | 4.563 | 4.432 – 4.699 | **<0.001** | 5.317 | 5.209 – 5.426 | **<0.001** | 0.820 | 0.802 – 0.837 | **<0.001** | 2.967 | 2.864 – 3.073 | **<0.001** | 3.254 | 3.147 – 3.365 | **<0.001** |
| Word count | 1.000 | 1.000 – 1.000 | **<0.001** | 1.000 | 1.000 – 1.000 | 0.455 | 1.001 | 1.001 – 1.001 | **<0.001** | 0.001 | 0.001 – 0.001 | **<0.001** | 1.000 | 1.000 – 1.001 | **0.001** | 1.001 | 1.001 – 1.001 | **<0.001** |
| Field [formal & natural] | 0.878 | 0.853 – 0.905 | **<0.001** | 0.991 | 0.940 – 1.045 | 0.739 | 1.089 | 1.050 – 1.129 | **<0.001** | -0.555 | -0.580 – -0.529 | **<0.001** | 0.265 | 0.252 – 0.279 | **<0.001** | 0.316 | 0.301 – 0.332 | **<0.001** |
| Field [medical & health] | 1.074 | 1.040 – 1.108 | **<0.001** | 0.572 | 0.541 – 0.606 | **<0.001** | 0.688 | 0.661 – 0.715 | **<0.001** | -0.437 | -0.464 – -0.409 | **<0.001** | 0.440 | 0.416 – 0.465 | **<0.001** | 0.528 | 0.501 – 0.557 | **<0.001** |
| Author number | 1.014 | 1.013 – 1.014 | **<0.001** | 1.004 | 1.003 – 1.004 | **<0.001** | 1.004 | 1.003 – 1.004 | **<0.001** | 0.002 | 0.001 – 0.002 | **<0.001** | 1.008 | 1.007 – 1.008 | **<0.001** | 1.012 | 1.011 – 1.012 | **<0.001** |
| Institutional diversity | 0.923 | 0.912 – 0.934 | **<0.001** | 0.990 | 0.969 – 1.011 | 0.338 | 0.993 | 0.979 – 1.008 | 0.361 | 0.072 | 0.061 – 0.083 | **<0.001** | 1.327 | 1.296 – 1.358 | **<0.001** | 1.224 | 1.198 – 1.252 | **<0.001** |
| Positivity | 1.001 | 1.001 – 1.001 | **<0.001** | 1.006 | 1.006 – 1.007 | **<0.001** | 1.007 | 1.007 – 1.007 | **<0.001** | 0.001 | 0.000 – 0.001 | **<0.001** | 1.000 | 0.999 – 1.000 | 0.457 | 1.000 | 1.000 – 1.001 | 0.525 |
| Promotional language * Year | 0.996 | 0.995 – 0.997 | **<0.001** | 0.999 | 0.998 – 1.001 | 0.251 | 0.996 | 0.995 – 0.997 | **<0.001** | 0.001 | 0.001 – 0.002 | **0.002** | 1.005 | 1.003 – 1.007 | **<0.001** | 1.006 | 1.004 – 1.008 | **<0.001** |
| Observations | 136612 | | | 136612 | | | 136612 | | | 64690 | | | 64690 | | | 64690 | | |

*Note*. *b* = unstandardized regression coefficient; IRR=Incidence Rate Ratio; CI = 95% Confidence Interval. Citations: citation count per year; Views 180: the number of times the record has been accessed on the Web of Science platform (e.g. by clicking on the link to the publisher’s website or by saving it in a bibliographic management tool) in the last 180 days; Views 2013: the number of times the record has been accessed on the Web of Science platform (e.g. by clicking on the link to the publisher’s website or by saving it in a bibliographic management tool) since 2013, per year. Altmetric: Altmetric score; Tweets: number of mentions on Twitter/X; Posts: overall number of mentions in any type of online document (news, social media, policy documents etc.). Promotional language: share of promotional language in the abstract; Journal: PNAS is reference category; Year: publication year (mean centered); Word count: number of words in the abstract; Field: social and humanities is reference category; author number: number of authors; institutional diversity: number of listed affiliations divided by the number of authors, higher score indicates more collaborations across different institutions; Positivity: overall semantic positivity of the abstract, obtained using the Linguistic Inquiry and Word Count software (LIWC, 2007).

Supplementary Figure 2

*Predicted academic impact and public attention as a function of promotional language and publication year*


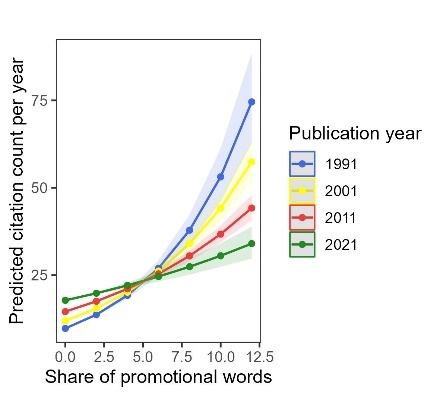

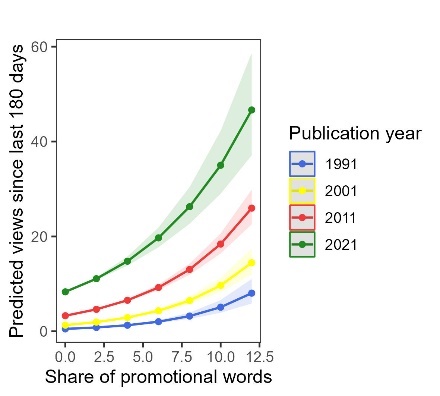

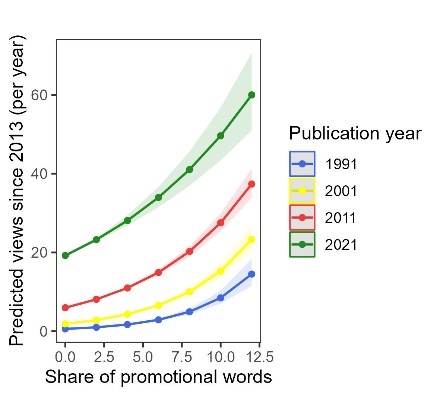

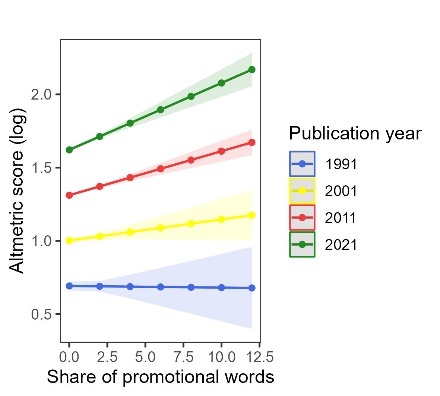

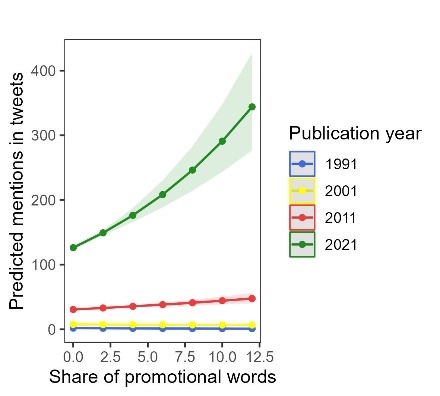

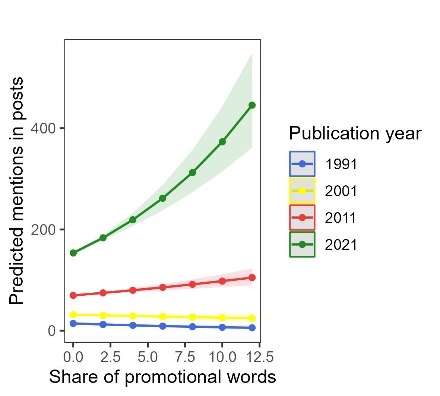


*Note.* The predicted values are shown for four selected years, starting with the first year of observation (1991) and continuing in four year increment .

# Correlations among impact indicators

Supplementary Table 11

*Means, standard deviations, and correlations among impact variables*

| Variable | *M* | *SD* | 1 | 2 | 3 | 4 | 5 |
| --- | --- | --- | --- | --- | --- | --- | --- |
|  |  |  |  |  |  |  |  |
| 1. Citation count per year | 14.96 | 31.53 |  |  |  |  |  |
|  |  |  |  |  |  |  |  |
| 2. Usage since last 180 days | 3.87 | 14.75 | .58*** |  |  |  |  |
|  |  |  | [.58, .58] |  |  |  |  |
|  |  |  |  |  |  |  |  |
| 3. Usage since 2013 per year | 7.08 | 22.96 | .60*** | .90*** |  |  |  |
|  |  |  | [.60, .61] | [.90, .90] |  |  |  |
|  |  |  |  |  |  |  |  |
| 4. Altmetric score | 1.43 | 0.83 | .25*** | .23*** | .26*** |  |  |
|  |  |  | [.24, .26] | [.22, .23] | [.25, .27] |  |  |
|  |  |  |  |  |  |  |  |
| 5. Mentions in tweets | 73.86 | 457.74 | .17*** | .10*** | .10*** | .27*** |  |
|  |  |  | [.16, .18] | [.09, .11] | [.09, .11] | [.27, .28] |  |
|  |  |  |  |  |  |  |  |
| 6. Mentions in blogposts | 109.49 | 588.66 | .20*** | .12*** | .12*** | .30*** | .99*** |
|  |  |  | [.19, .21] | [.11, .13] | [.11, .13] | [.29, .31] | [.99, .99] |
|  |  |  |  |  |  |  |  |

*Note.* ****p* < .001. Values in square brackets indicate the 95% confidence interval for each correlation.

# List of the subject fields

Formal and natural sciences: mathematical sciences, physical sciences, chemical sciences, earth and environmental sciences, biological sciences, agricultural and veterinary sciences, information and computing sciences, engineering and technology sciences.

Medical and health sciences: medical and health sciences

Social sciences & humanities: environment and design sciences, education sciences, economics, commerce and management sciences, studies of human societies, psychology and cognitive sciences, law and legal sciences, creative arts and writing sciences, language, communication and culture sciences, history and archaeology sciences, philosophy and religious sciences.

# References

1 Millar, N., Batalo, B. & Budgell, B. Trends in the use of promotional language (hype) in abstracts of successful national institutes of health grant applications, 1985-2020. *JAMA network open* **5**, e2228676-e2228676 (2022).
